# Recent Progress on the Long‐Term Stability of Perovskite Solar Cells

**DOI:** 10.1002/advs.201700387

**Published:** 2018-02-22

**Authors:** Qingxia Fu, Xianglan Tang, Bin Huang, Ting Hu, Licheng Tan, Lie Chen, Yiwang Chen

**Affiliations:** ^1^ College of Chemistry Nanchang University 999 Xuefu Avenue Nanchang 330031 P. R. China; ^2^ Jiangxi Provincial Key Laboratory of New Energy Chemistry/Institute of Polymers Nanchang University 999 Xuefu Avenue Nanchang 330031 P. R. China

**Keywords:** degradation, mechanisms, perovskite solar cells, stability

## Abstract

As rapid progress has been achieved in emerging thin film solar cell technology, organic–inorganic hybrid perovskite solar cells (PVSCs) have aroused many concerns with several desired properties for photovoltaic applications, including large absorption coefficients, excellent carrier mobility, long charge carrier diffusion lengths, low‐cost, and unbelievable progress. Power conversion efficiencies increased from 3.8% in 2009 up to the current world record of 22.1%. However, poor long‐term stability of PVSCs limits the future commercial application. Here, the degradation mechanisms for unstable perovskite materials and their corresponding solar cells are discussed. The strategies for enhancing the stability of perovskite materials and PVSCs are also summarized. This review is expected to provide helpful insights for further enhancing the stability of perovskite materials and PVSCs in this exciting field.

## 1 Introduction

With the problem of the prospective reserves of fossil fuels and the environmental pollution caused by the consumption process, the fossil energy cannot meet the needs of sustainable development in the future.^1^ Therefore, it generates a strong demand for implementation of clean and renewable energy. Solar energy is an inexhaustible green energy source which provides over 1000 times of power that the entire planet requires, while photovoltaic (PV) technology provides an ideal and clean route to be pursued. However, high cost and poor flexibility of silicon solar cells limit their future development.^2^ Furthermore, low‐cost, environment‐friendly new solar cells become the focus of widespread concern. Among all the PV technologies, the perovskite solar cells (PVSCs) have attracted much attention^3^, ^4^, ^5^, ^6^, ^7^, ^8^, ^9^, ^10^, ^11^ and considered to be a major discovery in the field of photovoltaics.^12^ The current highest certified power conversion efficiency (PCE) of PVSCs has reached 22.1%.^13^

**Figure 1:**
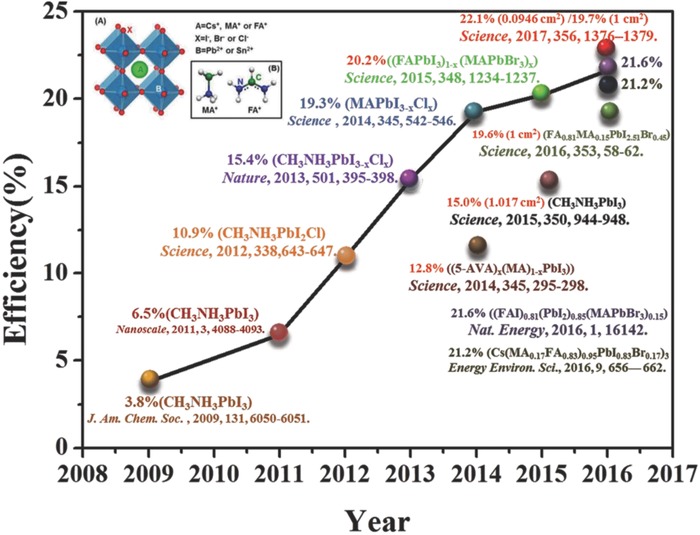
PVSCs power conversion efficiency chart from 2008 to 2017. The PCE highlighted with red circle is certified by an independent accredited test center.

Although PVSCs have been intensively studied in solar cells recently, these materials were first discovered by Weber 35 years ago. The 3D structure of perovskite materials was applied by Mitzi to transistors and light‐emitting diodes.^14^ In 2009, Kojima et al. put forward the first application of perovskite materials into quantum dots.^12^ The PCEs of 3.1 and 3.8% based on CH_3_NH_3_PbBr_3_ and CH_3_NH_3_PbI_3_ perovskite materials with TiO_2_ in the visible range were obtained, respectively. Park and co‐workers further significantly enhanced the PCE to 6.5% in 2011.^15^ Then MAPbI_3−_ *_x_*Cl*_x_* perovskite deposited on the mesoporous Al_2_O_3_ film that was deposited on a compact TiO_2_ layer to demonstrate a PCE of 10.9% upon contacting 2,2′,7,7′‐tetrakis(*N*,*N*‐di‐*p*‐methoxyphenylamine)‐9,9′‐spirobifluorene (spiro‐MeOTAD) as hole transport layer (HTL), which implied that perovskite materials can transport the electrons by itself without electron transporting materials.^16^ And then a remarkable progress of PVSCs was made with a certified PCE reaching 16.2%.^17^ In April 2014, Yang and co‐workers reported that the PCE of PVSCs has risen to 19.3%.^18^ The certified PCE of PVSCs with active area of >1 cm^2^ achieved 15.0% in 2015,^19^ and a further improvement in PCE of 1 cm^2^ device was achieved to 19.6%.^20^ With continuous efforts, a certified recording PCE of 22.1% was shown on the National Renewable Energy Laboratory best efficiencies chart in 2016 (**Figure** **1**).

The development of PVSCs has focused on two important issues with respect to practical applications. One is photoelectric conversion efficiency and the other is stability. The PCE of PVSCs has been increased from 3.1 to 22.1% in just a few years through materials design and device engineering based on the desired optoelectronic properties, while the PVSCs are now more competitive than those of traditional crystalline solar cells as a promising photovoltaic technology. Hence, the real challenge lies on the long‐term stability of PVSCs. PVSCs are prone to decay when exposed to humidity, heat, light, oxygen, and so on. At the same time, it is also important to explore device components, device structures, and interface properties. To be marketable within the industry, PVSCs must be able to operate continuously for close to 25 years in outdoor conditions. Long‐term stability tests for PVSCs without encapsulation should be over 500 h under 1 sun illumination^21^ and 1000 h under full sunlight.^22^ In this review, we summarized the recent progress in stability of PVSCs and discussed mechanisms of the device degradation from two aspects of perovskite layer and device structure. Various methods and outlooks on the future direction of perovskite materials and perovskite devices were also highlighted.

## 2 Mechanisms of Perovskite Degradation

The crystal structure of perovskites originated from the calcium titanium oxide mineral (CaTiO_3_). The general formula for the organic–inorganic halide perovskites is 3D ABX_3_, where A is CH_3_NH^+^ (MA^+^) or NH = CHNH^+^ (FA), Cs^+^, B is a metal ion (e.g., Pb^2^, Sn^2+^), X can be I^−^, Br^−^, or Cl^−^.^23^ In an ideal cubic‐symmetry structure, the B and X ions form BX_6_^4−^ octahedral, and A cation is located in the cavity between four BX_6_^4−^ octahedra and X is surrounded by 12 nearest neighbors (Figure 1). Rapid decomposition of PVSCs was found when exposed to prolonged humidity, heat, light, oxygen, etc.^6^, ^24^, ^25^ One of the major influence factors of degradation is the moisture. Therefore, the preparation of PVSCs should be in a humidity relatively low level of <1% atmosphere.^6^ Niu and co‐workers have presented a series of chemical reactions considered responsible for the degradation of CH_3_NH_3_PbI_3_ in moisture in the following Equations (1)–(4), 26

(1) $${\mathrm{CH}}_{3}{\mathrm{NH}}_{3}{\mathrm{PbI}}_{3}\;(s)\;\;⇄\;{\mathrm{PbI}}_{2}\;(s)\;\;+\;\;{\mathrm{CH}}_{3}{\mathrm{NH}}_{3}I\;(\mathrm{aq}.)$$

(2) $${\mathrm{CH}}_{3}{\mathrm{NH}}_{3}I\;(\mathrm{aq}.)\;\;⇄\;\;{\mathrm{CH}}_{3}{\mathrm{NH}}_{2}^{+}\;\;+\;\;\mathrm{HI}\;(\mathrm{aq}.)$$

(3) $$4\mathrm{HI}\;(\mathrm{aq}.)\;\;+\;\;O_{2}\;\;⇄\;\;I_{2}(s)\;\;+\;\;2H_{2}O$$

(4) $$2\mathrm{HI}\;(\mathrm{aq}.)\;\;⇄\;\;H_{2}\;+\;I_{2}(s)$$

Therefore, moisture degradation of perovskite is related with the MAPbI_3_ transforming to MAI salt and metal halides, while removal of metal halide can also accelerate perovskites device degradation. Walsh and co‐workers demonstrated that once the perovskite was exposed to H_2_O that was regarded as a Lewis base, CH_3_NH_3_PbI_3_ coordinated with H_2_O to form an intermediate [(CH_3_NH_3_^+^)*_n_*_−1_(CH_3_NH_2_)*_n_*PbI_3_][H_3_O], subsequently reducing the stability of perovskite.^27^ MAPbI_3_ undergoes serious degradation by the introduction of water molecules into the crystal structure, where they form weak hydrogen bonds to the highly hygroscopic methylammonium cations leading to bond dissociations between the crystal constituents.^25^, ^28^, ^29^ In addition, Philippe et al. declared that thermal treatment could induce the migration and interdiffusion of each component, resulting in the perovskite degradation.^30^ Heating at 100 °C for 20 min led to a significant reduction of I/Pb and N/Pb ratios. Moreover, instability in PVSCs also partly arises from UV light‐induced degradation due to a possible reaction between the TiO_2_ and perovskite.^31^ Such degradation of the perovskite is strongly related to their chemical components and corresponding crystal structures.

## 3 Perovskite Layer

A stable perovskite layer with high quality is crucial for the long‐term stability of PVSCs. This is because perovskite layer is very sensitive to external environment, consequently leading to the degradation of the PVSCs. In general, the stability of the perovskite layer depends on its chemical components, crystal structure, and the film quality of perovskites.

### 3.1 Crystal Structure of Perovskite Materials

The stability of PVSCs crystal structure ABX_3_ can be determined by a tolerance factor *t* and an octahedral factor *µ*, *t* = (R_A_ + R_X_)/{√2 (R_B_ + R_X_)}, *µ* = R_B_/R_X_, where R_A_, R_B_, and R_X_ are the ionic radii of the corresponding ions. Generally, to form a stable perovskite structure at room temperature, the value of *t* is between 0.81 and 1.11. When *t* is 0.9–1.0, it is expected to be ideal cubic perovskite, while distorted structures orthorhombic, rhombohedral, tetragonal, or hexagonal are likely to be formed when *t* is larger or smaller. At present, the value of *t* usually is 0.834 (R(CH_3_NH_3_^+^) = 0.18 nm, R(Pb^2+^) = 0.119 nm, and R(I^−^) = 0.220 nm) to the CH_3_NH_3_PbI_3_ perovskite. The *t* can be adjusted by the replacement or partial introduction of ions with different sizes to further obtain more stable cubic crystal structure.^3^, ^32^

#### 3.1.1 3D Perovskite

The organic cation (A) is a key part of the perovskite and can determine the perovskite crystal structure and its stability. When the slightly larger formamidinium ion FA (HC(NH_2_)_2_^+^) were replaced to MA^+^, *t* value increased to 0.99, which is higher than *t* = 0.91 for CH_3_NH_3_PbI_3_ (ion size: 2.53 Å for FA, 2.17 Å for MA, 1.19 Å for Pb^2+^, and 2.20 Å for I^−^) perovskite. The larger FA iron can also expand the lattice^33^ and change the tilt of the PbI_6_ octahedra,^34^ leading to a slight decrease of the band gap from ≈1.59 eV for MAPbI_3_ to ≈1.49 eV for FAPbI_3_.^35^ It was reported that the interaction of FA cation with surrounding PbI_6_ octahedra was stronger than that of MA cation due to higher probability to form hydrogen bonding.^36^ The mechanism for degradation of MAPbI_3_ under light was reported to the formation of hydroiodic acid (HI) by the released protons from MA cation with I^−^. However, protons released for FA cation is less than those released from MA cation due to resonance characteristics of FA cation via C—N bonds.^28^ Therefore, photostability is better for FAPbI_3_ than for MAPbI_3_. Compared to MAPbI_3_, FAPbI_3_ is not sensitive to high temperature. While MAPbI_3_ discolors in 30 min, FAPbI_3_ does not discolor even at 150 °C under ambient conditions.^37^ Unfortunately, FAPbI_3_ is found to show poorer humidity stability compared to MAPbI_3_.^38^ Black perovskite phase of FAPbI_3_ (α‐phase) was reported to be turned to yellow nonperovskite phase (β‐phase) in the presence of liquid interface at room temperature. Furthermore, FA^+^ cation tended to decompose into ammonia, which further degraded the moisture‐stability of FAPbI_3_.^39^

As presented in **Table** **1**, inorganic cesium (Cs) cation was partial substituted in FA site of FAPbI_3_ to achieve more stable trigonal black phase of FAPbI_3_. When the FAPbI_3_ and FA_0.9_Cs_0.1_PbI_3_ were exposed to continuous illumination (100 mA cm^−2^), the degree of degradation was severer for FAPbI_3_ film (85.9%) than for FA_0.9_Cs_0.1_PbI_3_ film (65.0%) after 19 h.^40^ The stability of FA_0.9_Cs_0.1_PbI_3_ film was much better than the pristine FAPbI_3_ film under 85% relative humidity at 25 °C in dark condition, due to more stabilized FA ions. The *t* value of Cs^+^ incorporated FA_1−_ *_x_*Cs*_x_*PbI_3_ perovskite was systematically investigated by Zhu and co‐workers.^41^ They found that FA_0.85_Cs_0.15_PbI_3_ with 15% Cs^+^ has a *t* value of 0.9 with a stable cubic phase. When FAPbI_3_ and FA_0.85_Cs_0.15_PbI_3_ thin films were exposed to a humid environment for 18 d, the absorbance value of FAPbI_3_ thin films decreased very fast while that of FA_0.85_Cs_0.15_PbI_3_ thin films remained constant. The PCE of the FA_0.85_Cs_0.15_PbI_3_ PVSCs showed almost no change while that of FAPbI_3_ decreased to about half the initial value when exposed to humid air for 400 h. Moreover, Park et al. also reported Cs/FA mixtures perovskite films enhanced the thermal and humidity stability of PVSCs, reaching a PCE of 16.5%. The improved structural stability was explained by that Cs was effective in assisting the crystallization of the black phase in FA perovskite due to entropic stabilization.^41^ Currently, a high‐quality perovskite films Cs*_x_*(MA_0.17_FA_0.83_)_(1−_ *_x_*_)_Pb(I_0.83_Br_0.17_)_3_ with Cs/MA/FA triple cation achieved PCE of 21.1% and output of 18% after 250 h under operational conditions.^42^ Moreover, Grätzel and co‐workers also developed a quadruple perovskite incorporating oxidation‐stable Rb into the Cs/FA/MA perovskite mixture. A high efficiency of 21.6% was obtained and the device retained 95% of its initial performance during 85 °C.^43^

**Table 1:** Performance summary and the stability of the perovskite solar cells with the different structures of perovskite materials. “Tolerance factor” was abbreviated as “*t*” in the stable

	Perovskite materials	*t*	PCE	Stability	Ref.
3D perovskite A cation	FAPbI_3_	0.987	16.0%	Thermally stable at 230 °C, light stable in humid conditions	41
	Cs*_x_*(MA_0.17_FA_0.83_)_(1−_ *_x_*_)_Pb(I_0.83_Br_0.17_)_3_	0.911	21.1%	85% for 250 h	42
	RbCsMAFA	0.870	21.6%	95% for 500 h, 85 °C	43
X cation	MAPb(I_1−_ *_x_*Br*_x_*)_3_	0.919	12.3%	Stable for 480 h, 55% humidity	6
	FA/MAPbI/Br	0.920	22.1%	>93% for 9360 h	14
	MAPbI_3−_ *_y_*Cl*_y_*	0.924	15.1%	95% for 720 h	51
	MAPb(I_1−_ *_x_*Br*_x_*)_3−_ *_y_*Cl*_y_*	0.925	11.1%	80% for 720 h	55
	MAPb(SCN)_2_I	0.834	8.3 %	Stable for 336 h, 95% humidity	57
	MAPbI_3−_ *_x_*(SCN)*_x_*	0.834	15.1%	>85% for 500 h, 70% humidity	59
2D perovskite	PEA_2_(MA)*_n_*_−1_Pb*_n_*I_3_ *_n_*_+1_	–	4.7%	Stable for 1140 h, 52% humidity	60
	(PEA)_2_(MA)_2_[Pb_3_I_10_]	–	15.3%	86% for 1344 h	61
	(PEI)_2_(MA)*_n_*_−1_Pb*_n_*I_3_ *_n_*_+1_	–	8.8%	90% for 500 h	62
	(BA)_2_(MA)*_n_*_−1_Pb*_n_*I_3_ *_n_*_+1_	–	4.0%	Stable for 1440 h, 40% humidity	63

Apart from A cation, the metal B atom in ABX_3_ is also critical for the structural stability of the perovskite materials.^3^, ^44^ The most commonly used B is Pb atom, but the toxic Pb faces potential barrier to commercialization. It is more favorable for considering nontoxic or environment‐friendly alternatives. It has been reported that using tin (Sn^2+^) with a smaller volume to replace Pb^2+^ caused a decline in the stability of the lattice.^5^, ^45^, ^46^ This is because due to the lack of the inert pair effect, Sn^2+^ is rapidly oxidized to more stable Sn^4+^ which is harmful to the structural stability of the perovskite.^47^ Tandem structure combination of FA_0.75_Cs_0.25_Sn_0.5_Pb_0.5_I_3_ with a wider‐band gap FA_0.83_Cs_0.17_Pb(I_0.5_Br_0.5_)_3_ perovskite materials has also been investigated as potential systems with the PCE of monolithic two‐terminal tandem PVSCs achieving 17.0%. Remarkably, such infrared‐absorbing perovskite cells exhibited excellent thermal and atmospheric stability. Obviously, we need further fundamental studies on the performance of Pb‐free perovskite and more advanced solar cell preparation techniques to develop high‐performance Pb‐free PVSCs in the future.

The highest reported stability of PVSCs with light absorbing MAPbI_3_ is 2000 h, which does not meet the requirement of the practical applications and commercialization. Incorporation of a small atomic radius of Br or Cl with similar properties into MAPbI_3_ perovskite has been found to be an effective approach to improve the device stability. Noh et al. tuned the stoichiometry of MAPb(I_1−_ *_x_*Br*_x_*)_3_ perovskites by substituting I^−^ with Br^−^.^6^ Interestingly, the MAPb(I_1−_ *_x_*Br*_x_*)_3_ (*x* = 0, 0.06) hybrid solar cells suffered from serious degradation after exposure to 55% humidity, whereas the PCE of perovskite with higher Br concentration maintained stable with no obvious degradation observed after 20 d. A low sensitivity to humidity of the cells based on higher Br content was associated with a reduced lattice parameter and a transition from tetragonal to cubic phase. When Br^−^ ions were introduced into the perovskite structure, the lattice parameters were changed, for instance, 5.921 for the MAPbBr_3_, 6.144 for the MABr/MAI 2:1, and 6.223 for the MABr/MAI 1:2. The change in the lattice parameter is mainly on account of the difference between the ionic radii of Br^−^ (1.96 Å) and I^−^ (2.2 Å).^48^, ^49^ The ionic radius of Br^−^ is smaller, which is also favorable for the formation of the cubic structure. Misra et al. reported the substitution of larger I atoms with smaller Cl atoms in MAPbI_3−_ *_x_*Cl*_x_* and stability of PVSCs improved. The authors showed that the improved stability can be related to its compact and stable structure, which led to a reduction of the lattice constant and a transition to a more stable cubic phase.^50^ Dai and co‐workers reported a layer‐by‐layer approach to fabricate MAPbI_3−_ *_y_*Cl*_y_* PVSCs with improved stability. The devices retained 95% of its initial PCE after storage in glovebox without any device encapsulation for 30 d.^51^ Later, chlorine incorporation was investigated to also improve the film crystallinity and provide a stable morphology as well as a better coverage in PVSCs.^52^ In addition, Cl incorporation was found to enhance the lifetime of the photoexcited species.^53^ However, experimental evidence for the presence of chloride in the perovskite films is less than 4%.^54^ In practical applications, the compatibility of Cl and iodide mixed halides and the basic contribution of chloride ion to stability need to be further solved. To evaluate the stability of MAPb(I_1−_ *_x_*Br*_x_*)_3−_ *_y_*Cl*_y_* PVSCs with different Br content, the PVSCs were stored in a glovebox under nitrogen atmosphere in the dark. The MAPbI_3−_ *_y_*Cl*_y_* PVSCs without Br took a PCE drop of 20% in 30 d, however with 50% Br content there was a notable increase of about 37% in PCE, due to the rearrangement of 3D perovskite.^55^

With similar chemical characteristics and ion radius to halogen anions, pseudohalogen anions can also affect the *t* value of perovskite structures.^56^ The thiocyanate anion (SCN^−^) is a stable pseudohalogen anion with similar properties to I^−^ ion. Liang and co‐workers fabricated MAPbI_3−_ *_x_*(SCN)*_x_* with a small amount of Pb(SCN)_2_ adding to a PbI_2_ solution. The partial substitution of I^−^ by SCN^−^ in the perovskite structure could remarkably improve the stability compared to the conventional iodide‐based perovskite.^57^ Xu and co‐workers developed a MAPb(SCN)_2_I perovskite by substituting I^−^ in MAPbI_3_ with two SCN^−^.^58^ The stability of MAPbI_3_ and MAPb(SCN)_2_I thin films exposed to 95% relative humidity air for several hours was investigated in the reflection spectrum. A substantially increased reflection value for MAPbI_3_ in 0.5 h indicated MAPbI_3_ perovskite quickly degraded under humidity. In contrast, the MAPb(SCN)_2_I reflection value increased slightly under the same humidity for 4 h. Moreover, the color of the MAPbI_3_ film was almost completely changed to yellow in air for 30 d, indicating the formation of PbI_2_, whereas no significant change was observed for MAPb(SCN)_2_I. When exposed to 95% relative humidity air, the PCE of MAPbI_3_‐based device decreased from 8.8 to 6.9% in 7 d and completely decomposed in 14 d, while that of the MAPb(SCN)_2_I‐based device only decreased from 8.3 to 7.4% in 14 d. Yan and co‐workers also prepared a stable MAPbI_3−_ *_x_*(SCN)*_x_* in humidity air.^59^ The average PCE of perovskite devices was 13.49%, and the maximum PCE exceeded 15%, even when the relative humidity kept 70% over 500 h. Calculations showed that the introduction of SCN^−^ groups into the perovskite lattice was thermodynamically stable. Moreover, strong ionic interactions existed between SCN^−^ and the adjacent Pb^2+^ ions as well as hydrogen bonds forming between SCN^−^ and MA^+^, which helped to improve chemical stability.

#### 3.1.2 2D Perovskite

**Figure 2:**
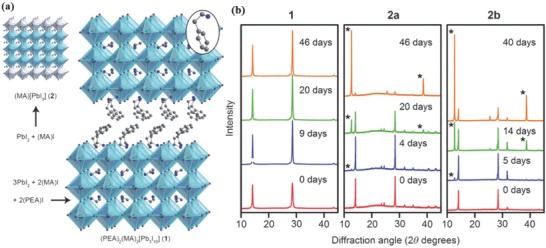
a) Crystal structures of the 3D perovskite MAPbI_3_ and the 2D perovskite (PEA)_2_(MA)_2_[Pb_3_I_10_]. b) PXRD patterns of (PEA)_2_(MA)_2_[Pb_3_I_10_] films (1), MAPbI_3_ formed from PbI_2_ (2a), and MAPbI_3_ formed from PbCl_2_ (2b), which were exposed to 52% relative humidity. Annealing of films of 2a (15 min) and 2b (80 min) were conducted at 100 °C prior to humidity exposure. Reproduced with permission.^60^ Copyright 2014, Wiley‐VCH.

In recent years, the application of 2D layered perovskite materials has been considered as a promising possibility material to improve the stability of PVSCs. The 2D perovskite materials were prepared by the insertion of larger cations, for example, C_6_H_5_(CH_2_)_2_NH_3_^+^ (PEA^+^),^60^ phenylethylammonium iodide (PEAI),^61^ PEI^+^,^62^ and CH_3_(CH_2_)_3_NH_3_^+^ (BA^+^)^63^ into the 3D MAPbI_3_ lattice. As presented in Table 1, Karunadasa and co‐workers demonstrated a 2D perovskite (PEA)_2_(MA)_2_[Pb_3_I_10_] (PEA = C_6_H_5_(CH_2_)_2_NH_3_^+^, MA = CH_3_NH_3_^+^) for the first time by integrating PEA^+^ into the 3D MAPbI_3_ perovskite.^60^ The crystal structures of MAPbI_3_ and (PEA)_2_(MA)_2_[Pb_3_I_10_] are compared in **Figure** **2**. High‐quality (PEA)_2_(MA)_2_[Pb_3_I_10_] perovskite films can be deposited through spin‐coating without thermal annealing. To compare the moisture stability of (PEA)_2_(MA)_2_[Pb_3_I_10_] and MAPbI_3_, both materials were exposed to 52% relative humidity. The (PEA)_2_(MA)_2_[Pb_3_I_10_] perovskite films kept the same X‐ray diffraction (XRD) patterns after the exposure for 46 d (Figure 2), indicating its good stability to moisture. However, the MAPbI_3_ perovskite films showed a new diffraction peak of PbI_2_ after just 4 d. The enhanced moisture stability was mainly attributed to the formation of high compact films. However, reducing the dimension of the inorganic components from the 3D structure causes an increase in the band gap to an estimated value of ≈2.1 eV for (PEA)_2_(MA)_2_[Pb_3_I_10_], resulting in lower device performance than 3D perovskite.

**Figure 3:**
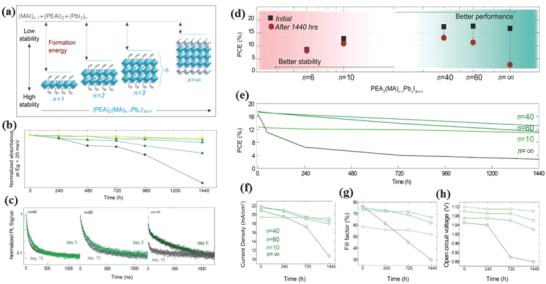
a) Unit cell structure of PEA_2_(MA)*_n_*_−1_Pb*_n_*I_3_ *_n_*_+1_. b) Relative absorption intensity near the absorption onset for different *n* values. c) PL decay data before and after 10 d storage. d) PCE of devices based on MAPbI_3_ and quasi‐2D perovskites before and after 1440 h storage. Stability of e) PCE, f) *J*_SC_, g) FF, and h) *V*_OC_ of perovskites with different *n* values. Reproduced with permission.^61^ Copyright 2016, American Chemical Society.

PEA‐based layered perovskite films were investigated by continuously tuning the dimensionality of perovskite compounds with mixing different stoichiometric quantities of PbI_2_, MAI, and PEAI to yield compounds with different layer (*n*) values in the series PEA_2_(MA)*_n_*_−1_Pb*_n_*I_3_ *_n_*_+1_.^61^ In this notation, the limit *n* = ∞ corresponds to the cubic 3D perovskite (CH_3_NH_3_PbI_3_) while other *n* values describe 2D (*n* = 1) or quasi‐2D (*n* > 1) perovskite structures (**Figure** **3**). The stability of the perovskite materials was investigated as a function of dimensionality by the absorbance and transient photoluminescence (PL) decay. After two months, the absorbance of 3D perovskite films at wavelengths longer than 500 nm significantly decreased while the quasi‐2D perovskites absorbance displayed no or only slight variation, indicating a significant improvement of stability with comparison to 3D perovskite (Figure 3). The PL decay traces showed dramatic changes for the 3D perovskite after 10 d storage in air whereas there was no observable variation in the charge‐carrier lifetime of quasi‐2D (*n* = 40, 60) perovskites. As a result, the device stability was remarkably improved. The PCE of the 3D perovskite dropped to 18% of its original PCE after eight weeks when stored in N_2_, nevertheless devices based on the quasi‐2D perovskites kept 74% (*n* = 60) and 86% (*n* = 40) of its initial PCE after the same time under a low‐humidity atmosphere. It was concluded that quasi‐2D perovskites possessed much higher formation energies as a result of the appreciable van der Waals forces that confer to improve stability compared to 3D perovskites. The additional organic cations with larger radius can actually protect the perovskite structure as a capping layer in quasi‐2D perovskite.

**Figure 4:**
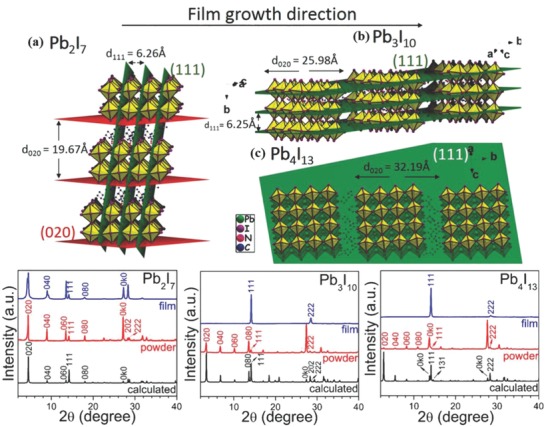
XRD patterns of a) (BA)_2_(MA)Pb_2_I_7_, b) (BA)_2_(MA)_2_Pb_3_I_10_, and c) (BA)_2_(MA)_3_Pb_4_I_13_ perovskite films, with the illustration of their respective diffraction planes. Reproduced with permission.^63^ Copyright 2015, American Chemical Society.

The fabrication of the 2D lead iodide perovskite (CH_3_(CH_2_)_3_NH_3_)_2_(MA)*_n_*_−1_Pb*_n_*I_3_ *_n_*_+1_ (*n* = 1–4) thin films was reported by employing a large alkylammonium ion CH_3_(CH_2_)_3_NH_3_^+^(BA^+^) (**Figure** **4**).^63^ The 2D perovskite films were formed in a self‐assembly fashion with preferentially oriented growth perpendicular to the substrate. It provided essential access to ultrasmooth and high‐surface‐coverage thin films. As expected, the (BA)_2_(MA)*_n_*_−1_Pb*_n_*I_3_ *_n_*_+1_ perovskites showed better moisture resistance compared to their 3D MAPbI_3_ analogues. The authors ascribed the moisture‐resistance of the (BA)_2_(MA)*_n_*_−1_Pb*_n_*I_3_ *_n_*_+1_ to the hydrophobicity of the long BA^+^ chain and the highly oriented and dense nature of the perovskite films, which prevented the direct contact of water molecules with the perovskite.

### 3.2 Morphology of the Perovskite Films

The perovskite film morphology has a strong effect on the stability of PVSCs. Many evidences have shown that the faster device degradation of PVSCs can be directly related to the perovskite grain size or grain boundaries.^64^, ^65^ Delicate control of their grain structures was considered to be an effective way to enhance not only the photovoltaic performance but also the stability of PVSCs.^66^ The preparation of high‐quality perovskite films is thus specially emphasized, including various solution processes and vacuum deposition method. In addition, the additives also play an important role in improving the crystallinity and morphology of perovskite layer deposition.

#### 3.2.1 Preparation Methods

In order to prepare high‐quality perovskite optical absorption layer, several representative methods have been developed, including the one‐step deposition from precursor solution, sequential solution deposition method, dual‐source thermal evaporation under vacuum, and vapor‐assisted solution process.

**Figure 5:**
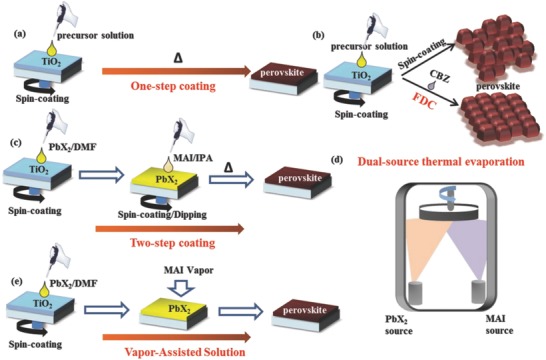
Schematic illustration of different preparation methods including a) one‐step method, b) fast deposition‐crystallization method, c) two‐step method, d) dual‐source thermal evaporation method, and e) vapor‐assisted solution process method procedures to deposit perovskite films.

*One‐Step Precursor Solution Deposition*: One‐step precursor solution deposition method is the most common method for the deposition of perovskite films due to its simplicity. In general, the perovskite precursor solution is prepared by mixing MAX and PbX_2_ (X: I^−^, Br^−^, Cl^−^) in γ‐butyrolactone (GBL), DMF, or dimethyl sulfoxide (DMSO) to a clear solution.^12^, ^15^ This precursor solution is spin‐coated on the substrate and then postdeposition annealed at 100–150 °C to complete the transformation of perovskite crystal films (**Figure** **5**). In one‐step method, the primary problem is that the naturally crystallized perovskite often tends to form homogeneous films. Such low‐quality perovskite film is prone to the rapid degradation of the PVSCs. Solvent composition,^67^ annealing, and processing temperatures^45^, ^68^ have a profound impact on the final film quality. Antisolvent engineering is employed to control the crystal growth kinetics, for example, toluene,^69^ chlorobenzene,^70^ or diethyl ether.^71^ Fast deposition crystallization (FDC) procedure is favorable to produce uniform and flat MAPbI_3_ perovskite film by one‐step deposition with the antisolvent engineering. In FDC procedure, a perovskite precursor solution is spin‐coated on the TiO_2_ layer, after a specific delay time, following by that a second solvent (chlorobenzene) is quickly added to the substrate to achieve the high‐quality perovskite thin films. The role of the second solvent is to rapidly reduce the solubility of CH_3_NH_3_PbI_3_ in the solvent and thereby promoting fast nucleation and growth of the crystals in the film (Figure 5).^72^ The large grain size formed by FDC method can reduce the grain boundaries of the perovskite films, leading to suppressing the perovskite from decomposition. This method has become the preferred method of deposition to yield all the subsequent efficiency records and the maximum PCE up to 21.6%.^73^

*Solution‐Processed Two‐Step Method*: Mitzi's group proposed two‐step sequential deposition in 1998 for the first time.^74^ In the typical sequential deposition process, a PbI_2_ layer was spin‐coated by 1 m PbI_2_ in DMF solution on the mesoporous TiO_2_ substrate, The dried PbI_2_ film was immersed in the isopropanol solution containing CH_3_NH_3_I, followed by rinsing with isopropanol solution to transform into perovskite films by annealing. As mentioned previously, dipping time and solution concentrations will be crucial to the morphology and optoelectronic property of the final perovskite films. The sequential deposition method is effective to monitor the PbI_2_ into TiO_2_ substrate, control the perovskite morphology, and improve the reproducibility of the device with comparison to one‐step deposition method^9^, ^75^ (Figure 5). However, in contrast to the mesoporous structure, the complete transformation of PbI_2_ to MAPbI_3_ with two‐step sequential deposition is generally a challenge for preparing planar perovskite films.^76^, ^77^ This is mostly because the morphology of the final perovskite film is largely determined by the PbI_2_ film and the reaction rate that is influenced by the diffusion of MAI into the PbI_2_ lattice. It has been reported that much thinner film with only about 200 nm can be obtained by two‐step method for the planar substrate. Once the thickness increases, it is difficult for CH_3_NH_3_I to penetrate deeper into the diffusion, resulting in appearing a large number of unreacted PbI_2_, which reduces the performance of the device.^77^

Two‐step spin‐coating technology was then proposed to solve the problem occurred by dipping process. Park and his co‐workers presented the “two‐step spin‐coating” approach, in which MAI solution was spin‐coated on the PbI_2_ film, instead of dipping the PbI_2_ film to the MAI solution, which showed to create nanocubic perovskite morphology.^78^ Perovskite crystal size and photovoltaic performance were found to be significantly influenced by the MAI concentration.^10^ Huang and his co‐workers developed the “interdiffusion” approach, where MAI was also interdiffused into the PbI_2_ film through spin‐coating the MAI solution, followed by a solvent‐vapor‐assisted annealing to control morphology.^79^ And now the best PVSCs efficiency prepared by the two‐step deposition method has been up to 22.1% with good stability.^13^

*Dual‐Source Thermal Evaporation*: Vapor deposition method was first reported to fabricate the uniform flat perovskite films under vacuum by Mitzi et al.^80^, ^81^ In 2013, Liu et al. applied the dual evaporation technique to fabricate the planar heterojunction MAPbI_3−_ *_x_*Cl*_x_* perovskite films.^8^ Due to the deposition rate detected by crystal oscillator, the thickness of the films can be controlled accurately. The perovskite film is much more uniform nanocrystal without any pinhole by the dual evaporation, compared to the solution process. The vapor‐deposition technique requires high vacuum and the complicated growth mechanism in vapor‐phase deposition, which is difficult to realize industrialization in the future^82^ (Figure 5).

*Vapor‐Assisted Solution Process*: Yang and co‐workers reported a new type of low‐temperature method vapor‐assisted solution process for the deposition of perovskite layers, combination of two‐step sequential deposition and thermal evaporation. The PbI_2_ films were spin‐coated on the compact TiO_2_, followed by CH_3_NH_3_I in the steam temperature of 150 °C and annealing in N_2_ for 2 h to form uniform perovskite grain structure with the PCE of 12.1% in this method (Figure 5). A simple and controllable method was presented to pursuit high‐quality perovskite films to effectively avoid the high reaction rate in the dual vapor deposition and defects generated in the two‐step sequential deposition.^9^ The perovskite films prepared by this method showed a better crystallization, high surface coverage, and small surface roughness,^83^ which is beneficial to improve the stability of perovskite. However, the gas–solid reaction typically requires tens of hours for the complete conversion. At present, the PVSCs prepared by this method have taken a maximum efficiency of 20.5% of PVSCs and the certified PCE of 19.6% with an aperture area exceeding 1 cm^2^.^20^

#### 3.2.2 Additives

Recently, incorporation of additives in the perovskite solution process has become a simple way to improve the film crystallinity and the stability,^84^ including polymer,^85^, ^86^ ammonium salt,^87^ hydrochloric acid,^88^ organic materials,^89^ inorganic acids,^88^ solvents,^71^, ^90^ MA/EtOH,^91^ ionic‐liquid additive of methylammonium acetate (MAAc), a molecular additive of thio‐semicarbazide (TSC),^92^ etc., as presented in **Table** **2**. Zhao et al. designed a novel perovskite solar cell architecture fabricated based on a long‐chain hygroscopic polyethylene glycol (PEG) polymer‐scaffold structure.^93^ The homogeneous morphology of the polymer‐scaffold perovskite films was successfully achieved with a highest PCE of 16% and high reproducibility. More importantly, the PEG scaffold perovskite significantly showed much better performance in stability test, retaining high PCE for up to 300 h in 70% relative humidity environment. With the strong interaction between MAI and the excellent hygroscopicity of the PEG molecules, the PEG scaffold perovskite devices demonstrated a self‐healing effect and the good long‐term stability. Nakamura and co‐workers fabricated PVSCs with a polar polymer polyvinylpyrrolidone (PVP) as an additive to control the crystal size and stability.^86^ Since the O atoms form hydrogen bonds with H atoms in the perovskite, the polymer PVP can wrap up the small perovskite crystals and prevent device deterioration under air atmosphere. Therefore, the stability of the PVP‐doped devices was further increased.

**Table 2:** Performance summary and the stability of the perovskite solar cells with the respective additives

Additives	Materials	PCE	Stability	Ref.
Polymer	PVP	12.7%	81.2% for 820 h	86
	PEG	16.0%	Stable for 300 h, 70% humidity	93
Organic halide salts	5‐AVA	12.8%	Stable for more than 1000 h in humid air	22
	FEAI	18.0%	92% for 2880 h	95
	EAI	10.7%	80% for over 360 h	96
	4‐ABPACl	16.7%	90% for 168 h, 45 °C	97
Inorganic acids	HCl	16.9%	95% for 720 h	98
Ionic liquid	MAAc + TSC	19.19%	Over 80% for 500 h, 85 °C	92

Chu and co‐workers investigated alkali metal halides (KCl, LiCl, NaCl) into the perovskite precursor solution as additives to obtain significant improvements in the perovskite morphology.^94^ Due to larger crystallites and decreasing grain boundaries by salt ions, the perovskite device stability prepared with the alkali metal halides additive was significantly improved. Alkylcarboxylic acid ω‐ammonium additives have also been used as templates for enhancing perovskite nucleation or crystal growth. All of the alkylcarboxylic acid ammonium additives were incorporated into the perovskite lattice and stuck between neighboring perovskite layers. Han and co‐workers first reported that 5‐aminovaleric acid cations (5‐AVA^+^) can be used as a template for the preferential crystal growth of MAPbI_3_ in the mesoporous oxide host by forming hydrogen bonds between its COOH and NH_3_^+^ groups and I^−^ ions from the PbI_6_ octahedra. The COOH group of 5‐AVA coordinated with mesoporous TiO_2_ and ZrO_2_ and the NH_3_^+^ ions on the terminal of 5‐AVA^+^ served as nucleation sites for the formation of the perovskite crystal 5‐AVA^+^.^22^ The PVSCs with 5‐AVA^+^ modified perovskite worked stably for more than 1000 h in humid air and under full sunlight. Nazeeruddin and co‐workers reported 1,1,1‐trifluoro‐ethyl ammonium iodide (FEAI) additive in MAPbI_3_ perovskite to show a better morphology and greatly enhance the stability of the perovskite.^95^ The author indicated the improved stability may be due to the formation of 1D 2H perovskite. Cheng and co‐workers tried to use ethyl‐ammonium iodine (EAI) as an additive in the perovskite precursor solution to disclose that a small amount of EAI could significantly affect the perovskite film morphology and the perovskite stability.^96^ The highest PCE of 18% was achieved with 3 mol% FEAI additive into the perovskite precursor solutions with improved environmental stability and enhanced moisture resistance. Grätzel and co‐workers used phosphonic acid ammonium additives (4‐ABPACl) forming the crosslinking of CH_3_NH_3_PbI_3_ perovskite via strong hydrogen bonding of the —PO(OH)_2_ and —NH_3_^+^.^97^ The robust perovskite can simultaneously improve the device efficiency and stability. The encapsulated PVSCs were continuously illuminated under 10 mW cm^−2^ ultraviolet‐filtered simulated sunlight at 45 °C for one week with performance of CH_3_NH_3_PbI_3_‐ABPA retaining 90%.

Xu and co‐workers fabricated highly smooth and complete coverage perovskite films by involved hydrochloride to perovskite films process with large crystalline grains. The authors proposed that the formation of Pb—I—Pb is much more difficult when chloride is coordinated to lead, leading to a slow nucleation and growth. When hydrochloride gas was dissolved in the perovskite precursor solution, the coordination tended to form chloride coordinated ions due to the high affinity between lead and chloride. Therefore, addition of hydrochloride enabled the improvement of perovskite film morphology with a large grain size. Moreover, the devices were highly air‐stable and the 95% of the initial PCE (16.9% certified) remained after 30 d storage in air at room temperature without encapsulation.^98^

## 4 Device Structure

### 4.1 Device Architectures

**Figure 6:**
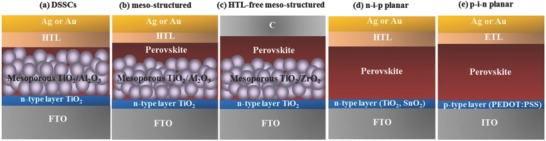
Schematic diagrams of the a) DSSCs, b) mesoporous structure (mesostructure), c) HTL‐free mesoporous structure, d) n‐i‐p planar structure, and e) p‐i‐n planar structures.

In 2006, CH_3_NH_3_PbI_3_ was employed as the dye sensitizers in dye‐sensitized solar cells (DSSCs) (**Figure** **6**). Therefore, PVSCs were developed from DSSCs. Now the structures of PVSCs with the perovskite as light absorbing material are mainly divided to mesoscopic structure and planar structure.^4^ These device structures also impact the device stability.

#### 4.1.1 Mesoporous Structure

Generally, the mesoporous structure consists of a transparent conductive oxide (TCO) cathode [fluorine doped tin oxide (FTO)], a compact electron transport layer (ETL) TiO_2_, a mesoporous metal oxide TiO_2_/Al_2_O_3_ as the transparent n‐type component filled with perovskites, followed deposited by perovskite layer, a HTL of spiro‐MeOTAD, and a metal anode (Au or Ag) (Figure 6). Kim and co‐workers investigated solid‐state PVSCs using a thick TiO_2_ scaffold and a solid‐state HTL called spiro‐OMeTAD on top of the perovskite layer instead of the liquid electrolyte in 2009 to achieve good performance of PVSCs for the first time.^12^ Then a 9.7% PCE was obtained for CH_3_NH_3_PbI_3_ absorber and a mesoporous TiO_2_ scaffold with the long‐term stability of PVSCs remarkably improved for over 500 h.^21^ Leijtens et al. attributed the dependence of device performance on the mesoporous TiO_2_ layer to a perovskite pore filling effect.^99^ Recently, Seok and co‐workers successfully fabricated the high‐performance and high stability of PVSCs containing formamidinium with multiple cations and mixed halide anions.^13^ Han and co‐workers adopted the HTL‐free fully printable PVSCs with the structure of FTO/TiO_2_/ZrO_2_/perovskite/C. The HTM‐free fully printable PVSCs demonstrated a certified PCE of 12.8% and the PVSCs were stable for >1000 h in ambient air under full sunlight^22^ (Figure 6). Priyadarshi et al. achieved a PCE of 10.75% with 70 cm^2^ area and demonstrated ambient stability of more than 2000 h with less than 5% reduction in efficiency by scalable printing processes.^100^ The association of the mesoporous layer and capping layer could assist in acquiring high efficiency with less hysteresis, but it also makes the perovskite deposition more difficult than the corresponding relevant PVSCs.^43^ Despite some work mentioned above, it remains controversial whether the presence of a scaffold is a prerequisite to improve the performance and the stability of PVSCs.

#### 4.1.2 Planar n‐i‐p and p‐i‐n Structure

Compared with the mesoporous structure of PVSCs, planar heterojunction solar cells have increased the flexibility optimization, providing the possibility for the development of the tandem solar cells, large‐scale industrial production, and the further study of the working mechanism for PVSCs. Planar heterojunction perovskite solar cells are usually composed of FTO conductive glass, compact barrier layer, perovskite layer, and hole conductor layer, which can be further divided into planar p‐i‐n and planar n‐i‐p structure. Figure 6 shows the general structure for planar n‐i‐p PVSCs of FTO/compact TiO_2_/perovskite/spiro‐OMeTAD/Au. In 2013, Ball et al. reported the first planer PVSCs with the PCE of 12.3%.^7^ The structure reduced the preparation temperature of the PVSCs. The planar CH_3_NH_3_PbI_3−_ *_x_*Cl*_x_* solar cells achieved an improved PCE of 15.4% by vapor‐phase deposition.^8^ The PCE of the planar structure has been promoted to 20.3% through morphology control and interface engineering.^101^ These results showed that similar device performance with the planar structure could be achieved as the mesoporous structure. Although the planar n‐i‐p perovskite solar cell typically exhibits enhanced *V*_OC_ and *J*_SC_ relative to a mesoscopic device processed with the same materials, the planar device usually holds more severe *J*–*V* hysteresis.

For another planar structure with the p‐i‐n configuration, the HTL is on top of the transparent conducting substrate, as shown in Figure 6. The most‐used HTL and ETL in the p‐i‐n structure is poly(3,4‐ethylenedioxythiophene):polystyrene sulfonate (PEDOT:PSS) and a fullerene derivative, for example, [6,6]‐phenyl C61‐butyric acid methyl ester (PC_61_BM) or [6,6]‐phenyl‐C71‐butyric acid methyl ester (PC_71_BM). The perovskite devices with the p‐i‐n structure provided the advantages of high efficiencies, lower temperature processing, flexibility, and negligible *J*–*V* hysteresis effects. The first planar p‐i‐n structure of PVSCs was delivered by Guo and co‐workers in 2013.^102^ Further development of the p‐i‐n device structure has expanded the selective contact options from organic to inorganic materials. For example, NiO_x_ and TiO_2_ layers have recently been used for the hole and electron selective contacts, respectively, which made the perovskite device distinct from its organic counterpart. Yang and co‐workers reported a solution‐processed lead halide perovskite solar cell with a maximum value of 16.1% based on p‐type NiO_x_ and n‐type ZnO nanoparticles as HTL and ETL, respectively.^103^ The device showed improved stability against water and oxygen degradation when comparing with the devices with organic charge transport layers. As a result, the PVSCs retained about 90% of their original efficiency after 60 d storage in air at room temperature.

In terms of the two kinds of device structures, ultimately all devices may adopt the simple structure of planar PVSCs configuration, representing a potentially important step in the path toward commercialization. However, at present, mesoscopic structures reached a certified stable PCE of 22.1%, which still holding a substantial lead over planar structures. The mesoporous TiO_2_ scaffold structure can promote collection of photogenerated charge carriers and enhance perovskite potential for future photovoltaics.^104^ Accordingly, the mesoscopic scaffold structure is boosted further with a compact perovskite capping layer.

### 4.2 Electron Transport Layer

**Figure 7:**
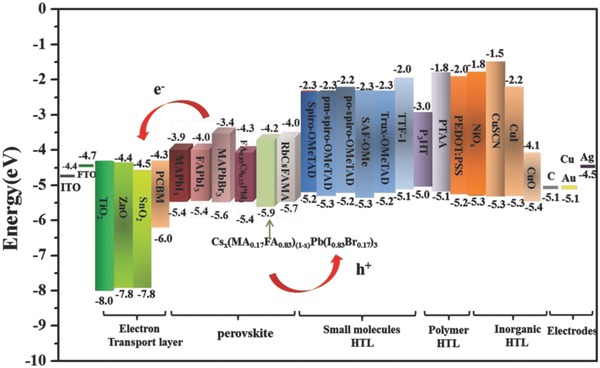
Scheme of the energy levels for perovskite materials and commonly used ETLs, HTLs, and the electrodes to the high performance and stability of PVSCs.

The ETL can be used to protect the perovskite layer out of degradation suffering from external environments. TiO_2_ has been used as the most frequently efficient inorganic ETLs in most PVSCs, owing to its favorable energy level, easy fabrication, and long electron lifetimes.^8^, ^10^ But the low electron mobility and relatively high density of electronic trap states below the conduction band (CB) of TiO_2_ increase the electron recombination rates, reducing the efficiency and stability under UV illumination of the device. Further results indicated that charge accumulation induced by ion migration in the perovskite layer could be the main cause of hysteresis.^105^ Hence, some alternatives were developed as ETLs to obtain efficient and better stability of PVSCs, as presented in **Figure** **7** and **Table** **3**. ZnO was known to have better electron mobility than TiO_2_.^106^ Additionally, the ZnO layer can be deposited easily without requiring such high‐temperature sintering step. However, other reports have also evidenced rapid decomposition of CH_3_NH_3_PbI_3_ when contact with ZnO.^107^ SnO_2_ have higher mobility than TiO_2_ and better stability with a band gap of above 3.6 eV, making it conceptually a more likely candidate in high‐performance PVSCs.^108^ Solution‐processed SnO_2_ nanoparticles have successfully been utilized as the ETL for PVSCs with a PCE of 20.5% with almost free of hysteresis.^109^ Recently, a new type of ETL La‐doped BaSnO_3_ (LBSO) with high electrical mobility was also employed as perovskite ETL.^110^ The best‐performing PSC exhibited a PCE of 21.2% and a very high stability below a 10% change for the PCE after 1000 h of full‐Sun illumination.

**Table 3:** The performance and stability of perovskite solar cells with different charge transport materials. In the table, perovskite is abbreviated as PVK, charge transport layers is abbreviated as CTL

CTL	Device structure	PCE	Stability	Ref.
ETL	ITO/ZnO/PVK/P_3_HT/Ag	11.4%	Stable for 1.3 h, 400 °C	107
	FTO/SnO_2_/PVK/spiro‐OMeTAD/Au	20.5%	Stable for 960 h, Dry air	108
	FTO/LBSO/PVK/PTAA/Au	21.2%	93.3% for 1000 h	110
	ITO/TiO_2_/Sb_2_S_3_/PVK/CuSCN/Au	5.2%	65% for 12 h	113
	FTO/TiO_2_/HOOC‐Ph‐SH/PVK/HS‐PhF_5_/spiro‐OMeTAD/Au	14.1%	>80% for 240 h, 45% humidity	114
	FTO/SnO_2_/C_60_‐N‐DBI/PVK/spiro‐OMeTAD/Au	18.3%	Stable for 656 h	124
	FTO/NiMgLiO/PVK/G‐PCBM/CQDs/Ag	15.6%	Stable for over 500 h, 85 °C	125
	FTO/NiMgLiO/PVK/PCBM/TiNbO_x_/Ag	16.2%	>90% for 1000 h light soaking	111
HTL	ITO/TiO_2_/PVK/TTF‐1/Au	11.03%	80% for 360 h, 40% humidity	131
	ITO/NiO_x_/PVK/ZnO/Al	16.1%	90% for 1440 h	103
	ITO/CuO_x_/PVK/C_60_/BCP/Ag	17.1%	>90% for 200 h	148
	FTO/TiO_2_/PVK/CuI/Au	6.0%	Stable for 2 h	149
	ITO/CuSCN/PVK/C_60_/BCP/Ag	16.6%	Stable for 28 h	150
	ITO/RGO/P/PVK/BCP/Ag	10.8%	>62% for 140 h	152
	FTO/TiO_2_/Al_2_O_3_/PVK/P3HT/SWNT/PMMA/Ag	15.3%	80% for 96 h, 80 °C	153
	FTO/TiO_2_/PVK/TSHBC/graphene/Au	14.0%	>90% for 240 h, 45% humidity	154
Electrodes	ITO/PTAA/PVK/C_60_/BCP/Cu	20.7%	98% for 816 h, 55% humidity	156
	FTO/TiO_2_/PVK/C	9.1%	Stable for over 2000 h	157
	FTO/c‐TiO_2_/meso‐TiO_2_/ZrO_2_/PVK/carbon	10.75%	95% for 2000 h	100

Since the TiO_2_‐based devices suffered from the degradation upon UV illumination, doping is an effective way to modify the electrical properties of TiO_2_ and improve the stability of PVSCs. Han and co‐workers successfully fabricated large‐size (>1 cm^2^) PVSCs with n‐doped (n^+^) TiO_x_ matrix with Nb^5+^ ions to improve rapid carrier extraction by avoiding pinholes and cracks over large areas. The result revealed that PVSCs maintained 97% of the initial PCE after 1000 h of light soaking with the Ti(Nb)O_x_ layer protecting the perovskite from intrusion of humidity.^111^ Al‐doped TiO_2_ could remove oxygen defects from the TiO_2_ lattice, leading to the improvement of the performance and enhancement in stability.^112^ Apart from doping, interface modification of TiO_2_ is also helpful to improve the device stability upon UV light. Ito et al. inserted a Sb_2_S_3_ layer between the TiO_2_ and CH_3_NH_3_PbI_3_ interface to block the UV‐induced photocatalysis and reduce the degradation of perovskite. While the control device almost did not work after 12 h exposure to light, with TiO_2_/Sb_2_S_3_ as ETL, PCE still maintained 65% of the initial value after the 12 h. This result again confirmed that UV‐induced reaction at the TiO_2_/CH_3_NH_3_PbI_3_ interface was partly responsible for the degradation of the perovskite.^113^ In addition, other efficient modifiers have also been reported, including carboxylic acid–thiol ligands (HOOC—Ph—SH),^114^ fullerene derivatives PCBDAN,^115^ ionic‐liquid,^116^ LiSPS,^117^ cesium chloride,^118^ and polyoxyethylene.^119^

Fullerene and its derivatives are the most widely used n‐type materials for organic ETLs, such as PC_61_BM, ICBA, and PC_71_BM.^102^, ^120^, ^121^ They are ideal candidates as efficient ETLs because of their low‐temperature fabrication, suitable energy level alignment, and decent electron mobility.^102^, ^120^, ^122^ Unfortunately, they possess the low electron mobility and photochemical instability.^123^ Further improvement in the PCE and stability of PVSCs was achieved by doping or modifying this type of ETLs. Snaith and co‐workers demonstrated the n‐doping of the electron accepting layer C_60_ to MAPbI*_x_*Cl_3−_ *_x_* and FA_0.83_Cs_0.17_Pb(I_0.6_Br_0.4_)_3_ PVSCs. The 4‐(1,3‐dimethyl‐2,3‐dihydro‐1H‐benzimidazol‐2‐yl)‐N,N‐diphenylaniline (N‐DPBI) dopant provided a higher conductivity and tuned the surface wettability of the C_60_ films. The devices achieved a PCE of 18.3% and long‐term stability owing to the reduced number of surface defects and filling of trap states with higher electron density.^124^ Yang and co‐workers deposited a nanostructured carbon layer acting as an ions/molecules blocking and electron extraction layer, containing N‐doped graphene, the fullerene derivative phenyl‐C61‐butyric acid methyl ester (PCBM), and carbon quantum dots (CQDs) for high efficiency and long‐term stability, which exhibited a stable efficiency of over 15% during thermal aging test at 85 °C for 500 h or light soaking under air mass 1.5 global (AM 1.5 G) illumination for 1000 h.^125^ Huang and co‐workers reported an insulating tunneling layer of polystyrene (PS), Teflon, and polyvinylidene‐trifluoroethylene copolymer (PVDF‐TrFE) interpolated between the perovskite and the electron collection layer in PVSCs can reduce charge recombination and greatly enhanced water stability of PVSCs.^126^

### 4.3 Hole Transport Layer

The HTL is also vital to prepare highly efficient and stable PVSCs. As a whole, the HTL can be divided into four families: small molecule, polymeric, inorganic, and carbon.^127^ Small molecules offer the advantages of high purity and reproducibility. Among these small molecules HTL, spiro‐MeOTAD is the most commonly used small molecule HTL.^128^ Remarkable efforts have been made to over 21% the PCE exceeding with spiro‐MeOTAD as the HTL. However, due to the relatively low hole mobility and poor conductivity, doping of lithium bis(trifluoromethanesulfonyl)imide salt (Li‐TFSI) and 4‐*tert*‐butylpyridine (TBP) is an essential step to enhance the hole mobility and suppress charge recombination.^129^ Furthermore, Li‐TFSI also acts as a counterion in this process to facilitate the oxidative reaction between spiro‐OMeTAD and O_2_.^130^ However, Li‐TFSI has aggravated the degradation of PVSCs due to its hygroscopic nature and the additional doping materials.^131^ Recently, doping‐free HTLs are fabricated with the high performance and good stability, such as tetrathiafulvalene derivative (TTF‐1),^131^ SAF‐OMe,^132^ 3,6‐di(2H‐imidazol‐2‐ylidene)cyclohexa‐1,4‐diene derivatives (DIQ‐C6 and DIQ‐C12),^133^ BTPA‐TCNE,^134^ etc. Chen and co‐workers present a new structural design of hole‐transporting material, Trux‐OMeTAD, which was designed by introducing triarylamine and aliphatic side chains onto the C_3h_ Truxene core with high mobility and suitable surface energy. As an effective HTL without the need of extra doping process, the excellent PV performance was achieved with a PCE of 18.6% and better stability.^135^

Compared to 3D structural spiro‐MeOTAD, conducting polymer materials own higher hole mobility and film‐forming property, constituting a major class of organic HTL in PVSCs. PEDOT:PSS is the generally adopted HTL.^136^ However, this hygroscopic nature of PEDOT:PSS limits the long‐term stability of inverted PVSCs.^137^ Doping with nanoparticles or conductive oxides, for example polyethylene oxide (PEO),^138^ molybdenum oxide (MoO_3_),^139^ graphene oxide (GO)^140^ is a commendable option for tuning the properties of PEDOT:PSS. More importantly, employing polytriarylamine (PTAA)^13^, ^141^ and poly(3‐hexylthiophene‐2,5‐diyl) (P3HT)^142^ to substitute PEDOT:PSS has been tested to dramatically improve the device stability. Bi et al. fabricated CH_3_NH_3_PbI_3_ film with large crystalline grains on PTAA HTL which dramatically reduced charge trap density and enhanced the PCE to ≈18.1% for planar PVSCs with its higher hole mobility.^65^ Extensive efforts have been made to introduce various novel HTMs to PSCs and promising progress has been achieved. To date, one of the highest reported PCE (22.1%) also used PTAA and large‐grain size microstructures as HTL.^13^

Inorganic materials were explored to be good candidates as HTL owing to their intrinsically properties, such as NiO_x_,^103^, ^143^ CuO_x_,^144^ CuSCN,^145^ CuI,^146^ CuPc,^147^ etc. Compared to organic hole conductors, inorganic p‐type materials usually possess high chemical stability, hole mobility, low cost, and ease of synthesis. Bian and co‐workers introduced a facile solution‐processed CuO_x_ as HTL in the inverted planar p‐i‐n PVSCs,^148^ in which the stability test of the unencapsulated device with CuO_x_ film suggested superior performance than that of device with PEDOT:PSS layer. Kamat and co‐workers reported another copper‐based inorganic p‐type hole‐conductor CuI as the p‐type HTL in mesoscopic PVSCs,^149^ demonstrating an inexpensive, stable, solution‐processable inorganic hole conductor. The PVSCs with CuI showed a promising PCE of 6.0%. Another inorganic p‐type semiconductor CuSCN has been drawn into PVSCs. High‐quality CH_3_NH_3_PbI_3_ films were fabricated with the CuSCN layer as the HTL,^150^ while the MAPbI_3−_ *_x_*Cl*_x_* (CuSCN) films hold lower surface roughness and smaller interface contact resistance between the perovskite layer and the selective contacts, exhibiting PCE of over 18%. Furthermore, the PVSCs based on CuSCN presented higher reproducibility and stability.^151^

Carbon‐based HTL offers one of the more stable solutions even though the device efficiency seems to be not yet comparable to the organic HTLs. Seok and co‐workers developed reduced graphene oxide (RGO) at the first time to enhance conductivity and the stability of the PVSCs.^152^ Snaith and co‐workers reported polymer‐functionalized single‐walled carbon nanotubes (SWNTs) embedded in an insulating polymer matrix replacing the organic HTL material. The polymer‐functionalized SWNT layer plays a role of a strong protective layer on the perovskites against moisture ingress and also operated outstanding thermal stability compared to other organic HTL materials.^153^ In 2015, Zheng and co‐workers employed for the first time a functionalized nanographene (perthiolated trisulfur‐annulated hexa‐peri‐hexabenzocoronene, TSHBC) by doping with graphene sheet as the HTL to achieve efficient charge extraction from perovskite. And the author found that the hydrophobic thiols on the perovskite surface easily inhibited water molecules to attack perovskite film and significantly increased the stability of PVSCs. The stabilities of the devices with TSHBC and TSHBC/graphene layers were measured under AM 1.5 G illumination in about 45% humidity without any encapsulation. The devices based on TSHBC/graphene kept more than 90% of their original efficiency after 10 d. In contrast, the cells with spiro‐MeOTAD kept 20% of their efficiency under the same storage condition after 10 d, indicating the devices involving TSHBC exhibited significantly improved device stability.^154^

### 4.4 Electrodes

Metal electrodes are an important part in PVSCs for charge collection. The common metal electrodes are silver, aluminum, and gold in the PVSCs.^155^ With high cost and requiring a high‐vacuum evaporation process, Au is limited in its future application. Moreover, the diffusion of Au across the thick HTL was reported recently, which resulted in the degradation of the devices in several hours. Silver is a cheaper choice compared to gold, but a silver electrode in PVSCs is found to react with the halide ions in humid environment, forming silver halides such as silver chloride (AgCl) or silver iodide (AgI) and causing the degradation of PVSCs. In contrast, Huang and co‐workers reported copper (Cu) as the electrode material in PVSCs for long‐term stability with no direct reaction to perovskite.^156^ The device with Cu electrode achieved high performance with efficiency above 20% and retained 98% of the original efficiency after 816 h storage in ambient environment without encapsulation. Ma and co‐workers successfully prepared full solution‐processed low‐cost PVSCs with a low‐temperature carbon electrode, exhibiting advantageous stability (over 2000 h) in air in the dark without encapsulation.^157^ Han and co‐workers fabricated a fully printable PVSCs with C as the electrode, exhibiting excellent stability.^22^ Accordingly, the carbon electrode offered the possibilities to the future large‐scale and flexible fabrication process of PVSCs. Mhaisalka and co‐workers reported the fabrication of carbon‐based stable large‐area monolithic perovskite solar modules (PSMs) with active area 70 cm^2^.^100^ The large area cells showed a PCE ≈ 11% with an excellent stability over 2000 h in ambient conditions.

### 4.5 Encapsulation

For commercial solar cells, such as silicon solar cells, CIGS solar cells, etc., encapsulation methods are applied to improve the stability of the devices.^158^ Encapsulation can eliminate the interaction of perovskites with environmental molecules due to the sensitivity of perovskite materials to the outdoor environment. So far only a few studies have focused on the encapsulation of the devices. Leijtens et al. demonstrated that the CH_3_NH_3_PbI_3−_ *_x_*Cl*_x_* perovskite devices with a mesoporous Al_2_O_3_ scaffold were encapsulated with an epoxy resin and a glass coverslip in a nitrogen‐filled glove box.^159^ Their devices showed only a small decrease in photocurrent over 1000 h under continuous full spectrum AM 1.5 G solar illumination at 40 °C. Spiccia and co‐workers adopted a sealing technique from the OLED technology that effectively reduced the ingress of moisture into the cell at high temperature to improved stability of PVSCs.^160^ Yong and co‐workers introduced a hydrophobic polymer Teflon as polymer encapsulation to enhance stability of perovskite solar cell under ambient atmosphere conditions.^161^ The PCE of the Teflon‐PVSCs exhibited considerably stable performances retaining 95% of the initial PCE after 30 d. The PVSCs also showed stable behavior over a certain time while it was even immersed in water. This facile passivation process would make a synergy with a further encapsulation process, providing PVSCs much improved stability. To further improve the device stability against moisture and harsh environments, appropriate encapsulation with water resistance, UV light protection, and functional layers are definitely desired for circumventing the device performance decay and prolonging the life span of PVSCs for practical applications.

## 5 Conclusion and Prospects

Although the PCE exceeding 22% has been achieved in PVSCs with the unprecedented rapid development, commercialization of PVSCs will require the synergistic development of high‐efficiency, large‐area with long‐term stability as a competitive technology. However, the actual long‐term stability of PVSCs still lags behind their outstanding efficiency. Thus, the various causes of devices degradation need to be further explored in‐depth to achieve high performance and stable PVSCs. The stability of perovskite layer is usually optimized by compositional engineering of halides and cations, the application of 2D materials and additives. On the other hand, incorporating proper charge transport layers and electrodes or interfacial resistant agents into PVSCs is also considered as effective ways to maintain device stability as well as the efficiency of PVSCs.

Therefore, the ultimate challenge for PVSCs is to achieve stable devices and perovskite materials that can survive in an outdoor environment. For the perovskite layer, ions selection, doping, and crystal structure are promising to improve the perovskite layer stability. For example, FA/MAPbI*_x_*Br_3−_ *_x_* perovskite material is more appropriate for the high stability of 3D perovskite layer. In addition, 2D systems provide new interesting challenges and the possibilities to new physical properties, also regarding charge transport, making them currently gain more attention in the field. Apart from the perovskite materials, stable device structures, interfacial engineering to form a stable and efficient interface interaction, inert electrode and proper encapsulation are also crucial. To date, the PVSCs with the best stability were achieved with mesoporous structure of FTO/c‐TiO_2_/meso‐TiO_2_/perovskite/PTAA/Au. In our view, the further improved mesoporous device structure has a great practical application potential due to the simplicity and ease of fabrication with decent performance. However, deposition of the mesoporous layer by avoiding high‐temperature annealing process would make more convenient for fabricating large‐area and the long‐term stability of PVSCs. The commonly used TiO_2_ is sensitive to UV with presence of surface defects (oxygen vacancies) in TiO_2_, which leads to the degradation of PVSCs. Therefore, exploring stable novel ETLs to replace TiO_2_ is highly desirable. For instance, the solution‐processed SnO_2_ is the good candidate for the high‐performance electronic devices instead of TiO_2_. Furthermore, with regard to the HTLs, developing doping‐free, cheap organic material instead of expected Li salt‐doped spiro‐MeOTAD or developing the HTL‐free structure devices is very attractive. For the planar p‐i‐n PVSCs, some stable and efficient inorganic, such as NiO_x_ HTL is potential to replace PEDOT:PSS. Hence, fabricating PVSCs with reliable high efficiency and long‐term stability is a systematic and very multidisciplinary project, which requires contributions from several directions.

Much progress has been already made in the stability of the halide perovskite. Nevertheless, the underlying mechanism of the degradation remains far from being comprehensively understood. To realize the industrialization development of PVSCs, an in‐depth comparative understanding of the degradation mechanisms is essential for establishing efficient solutions to improve the PCE and the stability of PVSCs.

## Conflict of Interest

The authors declare no conflict of interest.
